# Preoperative Breast MRI and Histopathology in Breast Cancer: Concordance, Challenges and Emerging Role of CEM and mpMRI

**DOI:** 10.3390/diagnostics15233032

**Published:** 2025-11-28

**Authors:** Aikaterini-Gavriela Giannakaki, Maria-Nektaria Giannakaki, Dimitris Baroutis, Sophia Koura, Eftychia Papachatzopoulou, Spyridon Marinopoulos, Georgios Daskalakis, Constantine Dimitrakakis

**Affiliations:** 1 First Department of Obstetrics and Gynecology, Alexandra General Hospital, National and Kapodistrian University of Athens, 11528 Athens, Greece; dbaroutis@gmail.com (D.B.); sophia_koura@hotmail.com (S.K.); efipapaxatzopoulou@hotmail.com (E.P.); smarinopoulos@outlook.com (S.M.); gdaskalakis@yahoo.com (G.D.); 2 Medical School, Aristotle University of Thessaloniki, 54124 Thessaloniki, Greece; giannakakimarnek@gmail.com

**Keywords:** breast MRI, histopathology, mastectomy, tumor size, invasive lobular carcinoma, ductal carcinoma in situ, neoadjuvant therapy, AI radiomics, multiparametric MRI, contrast-enhanced mammography

## Abstract

**Background:** Preoperative breast MRI is widely used in surgical planning because of its high sensitivity. However, discrepancies with histopathology remain common and can affect tumor size assessment and treatment decisions. In addition, recent comparative studies have highlighted the growing role of contrast-enhanced mammography (CEM) and multiparametric MRI (mpMRI), both of which may improve specificity and accessibility compared to conventional MRI. **Methods**: A structured literature review was conducted in PubMed (2000–2025) according to PRISMA guidelines. Studies included if they evaluated preoperative breast MRI with histopathological correlation and reported sensitivity, specificity, or concordance outcomes. Data extraction focused on study design, patient and tumor characteristics, imaging methods, and clinical impact. **Results:** MRI demonstrates high sensitivity, particularly in detecting IDC and ILC. However, overestimation of tumor size remains a concern, particularly in ILC and high-grade DCIS, while underestimation is frequently observed after neoadjuvant therapy, especially in Luminal A tumors. Tumor size and stage significantly affect concordance, with advanced-stage tumors (T2–T3) showing better MRI-histopathology concordance than early-stage lesions (T0–T1). Specificity remains limited, particularly in DCIS and multifocal disease. Emerging evidence suggests that contrast-enhanced mammography (CEM) achieves comparable sensitivity with higher specificity, while multiparametric MRI (mpMRI) incorporating diffusion-weighted imaging (DWI) improves lesion characterization and prediction of treatment response. **Conclusions:** While MRI remains a valuable diagnostic tool for breast cancer, histopathological validation is essential to guide treatment decisions. Future research should focus on AI-enhanced imaging techniques, CEM and multiparametric MRI to improve concordance rates, reduce overdiagnosis and translate imaging advances into meaningful clinical outcomes.

## 1. Introduction

Breast cancer remains the most common malignancy in women and a leading cause of cancer-related death worldwide [1]. Beyond established genetic factors (BRCA1, BRCA2, TP53, CDH1, PTEN) and lifestyle contributors (obesity, alcohol consumption, hormone replacement therapy), accurate imaging remains central to both detection and treatment planning [2,3].

Conventional imaging modalities such as mammography have served as first-line diagnostic tools. However, they show limitations in sensitivity, particularly in women with dense breasts and in subtypes such as invasive lobular carcinoma (ILC) [4]. Magnetic resonance imaging (MRI) has emerged as a highly sensitive modality for breast cancer detection, staging, and preoperative planning. Multiple studies report its ability to delineate tumor extent more accurately than traditional techniques [5,6,7].

Despite its strengths, MRI has limitations. It often overestimates tumor size, especially in ductal carcinoma in situ (DCIS) and ILC, leading to more mastectomies without corresponding reductions in re-excisions or improved long-term outcomes [8,9].

Among invasive subtypes, the most common are invasive ductal carcinoma (IDC) and invasive lobular carcinoma (ILC). IDC typically forms discrete, mass-like lesions that correlate well with histopathologic findings, whereas ILC often shows diffuse, infiltrative growth patterns and subtle enhancement, leading to underestimation of tumor size and extent in MRI. These differences in morphology and imaging appearance largely explain the variable MRI-histopathology concordance observed across subtypes [10].

In neoadjuvant settings, MRI may often underestimate residual disease, raising concerns about its reliability in predicting complete pathological response. This underscores the importance of correlating MRI findings with histopathology, which remains the diagnostic gold standard [11,12,13,14].

Importantly, the diagnostic landscape is rapidly evolving. Contrast-enhanced mammography (CEM) and multiparametric MRI are increasingly adopted in clinical practice. Some studies suggest equivalent or superior specificity compared with MRI, at lower cost with broader accessibility [15,16,17,18]. Meanwhile, artificial intelligence (AI) and radiomics may offer novel avenues for extracting predictive biomarkers from imaging data, potentially bridging gaps between imaging and pathology [19,20].

Recent clinical data confirm that, although preoperative MRI may provide superior lesion detection, it does not consistently improve long-term outcomes. However, integrating AI-driven analytics, CEM, and mpMRI appear to demonstrate measurable gains in diagnostic accuracy and workflow efficiency. Across recent comparative studies, the specificity ranged between 75–88%, while mpMRI achieved size concordance rates of 82–90% with histopathology. Most included studies were performed on 1.5 T or 3 T scanners, with sample sizes typically between 100–350 patients, and represented diverse tumor subtypes including IDC, ILC, and DCIS, which partly explains observed heterogeneity in diagnostic performance. Comparative reports increasingly explore how these modalities influence surgical re-excision rates and cost-effectiveness, supporting their growing role in personalized breast oncology.

This trend is further supported by contemporary high-impact studies, which collectively demonstrate that integrating AI-driven mpMRI may improve histopathological concordance, predict therapeutic response, and enhance individualized treatment planning in breast cancer [21,22,23].

Considering these developments, a critical synthesis of MRI-histopathology concordance is warranted, not only to summarize existing knowledge but also to evaluate gaps, controversies, and comparative evidence with emerging modalities. This review aims to systematically appraise the diagnostic concordance between MRI and histopathology, compare MRI with CEM, and discuss the future integration of AI-enhanced imaging into breast cancer diagnostics.

## 2. Materials and Methods

### 2.1. Search Strategy

A comprehensive PubMed search (2000–2025) was performed using MeSH and free-text keywords: breast cancer, MRI, histopathology, concordance, contrast-enhanced mammography (CEM), and multiparametric MRI (mpMRI). Cross-database searches in EMBASE, Scopus, and the Cochrane Library yielded no additional eligible studies beyond those retrieved from PubMed. This review followed selected PRISMA 2020 principles for transparent reporting. However, it is a structured narrative review rather than a full systematic review or meta-analysis, and no protocol registration, pooled estimates, or meta-analytic statistical methods were performed.

### 2.2. Eligibility Criteria (PICOS)

- Population (P): Adult women with biopsy-proven breast cancer (IDC, ILC, DCIS).
- Index test (I): Preoperative breast MRI (conventional DCE-MRI or mpMRI including DWI ± MRS); comparative analyses with CEM when available.
- Comparator (C): Histopathology (surgical specimen or core biopsy) as the reference standard.
- Outcomes (O): Diagnostic performance (sensitivity and specificity), size concordance, effect on surgical management, neoadjuvant response (pCR) prediction.
- Study design (S): Prospective and retrospective cohorts, diagnostic accuracy studies, randomized clinical trials, systematic reviews and meta-analyses.

Exclusions: case reports, male breast cancer, non-peer reviewed items, no histopathology comparator, non-English.

### 2.3. Study Selection

Two independent reviewers screened all titles and abstracts for eligibility. Inter-reviewer agreement was substantial, and any discrepancies were resolved through discussion and consensus. In cases where consensus could not initially be reached, a third reviewer was consulted to ensure methodological consistency. Full-text articles were reviewed when abstracts did not provide sufficient information for inclusion or exclusion criteria. Priority was given to high-quality studies such as randomized clinical trials, systematic reviews, and meta-analyses. The process of study selection is illustrated in PRISMA flow diagram, which shows the number of records identified, screened, excluded, and included in the narrative synthesis.

### 2.4. Data Items Extracted

- Study characteristics (Author, year of publication, study design)
- Patient demographics (Sample size, age distribution)
- Tumor characteristics (Subtype, size, stage, grade, and multifocality)
- Imaging methods (Use of MRI, supplementary imaging techniques)
- Outcomes (MRI-histopathology concordance, sensitivity, specificity, correlation coefficients and impact on surgical management)

The methodological quality of the included studies was assessed using a simplified QUADAS–2 approach (’QUADAS–lite’), based on the information reported in each publication. Risk of bias was judged qualitatively as low, moderate, or high across domains of patient selection., MRI protocol, reference standard, and flow/timing. Applicability was evaluated with respect to clinical relevance to MRI-histology concordance and diagnostic accuracy of CEM or mpMRI (Table 1).

Quantitative synthesis was not performed because of heterogeneity in imaging parameters, patient populations, and outcomes metrics (sensitivity, specificity, concordance).

### 2.5. PRISMA Flow Diagram

The initial PubMed search (2000–2025) identified 7542 records. After removing 2710 duplicates, 4832 unique studies remained for screening. Following title and abstract review, 460 full-text articles were assessed for eligibility, of which 374 were excluded (most commonly due to absence of histopathological correlation, incomplete diagnostic data, or non-original design). Finally, 86 studies were included in the qualitative synthesis and 48 of these fulfilled all predefined inclusion criteria and were analyzed in depth. These encompassed investigations of conventional preoperative MRI-histopathology concordance as well as studies evaluating contrast -enhanced mammography (CEM) and multiparametric MRI (mpMRI).

**Figure 1:**
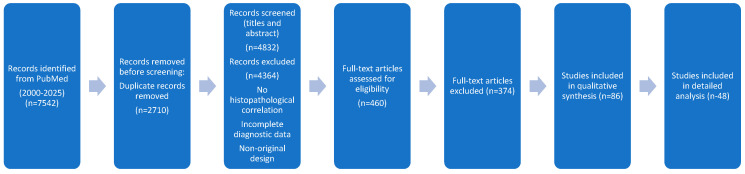
Flow Diagram.

The selection process is presented in a PRISMA flow diagram (Figure 1), summarizing the number of records identified, screened, excluded, and studies included in the final qualitative synthesis. The PRISMA 2020 Checklist related to this review is available in the Supplementary Materials.

### 2.6. Study Heterogeneity and Methodological Overview

Included studies demonstrated notable heterogeneity regarding imaging protocols, patient selection, and clinical context. Approximately two-thirds of the studies included employed 1.5 T MRI systems and one-third used 3 T scanners. Sample sizes varied widely, with a median of about 120 patients (range 45–520), reflecting the predominance of single-center retrospective cohorts. In terms of histologic subtype distribution, invasive ductal carcinoma (IDC) represented most cases (~60–70%), followed by invasive lobular carcinoma (ILC, ~15–20%) and ductal carcinoma in situ (DCIS, ~10–15%). About 40% of studies focused on patients receiving neoadjuvant systemic therapy, while 60% evaluated those undergoing upfront surgery. These proportions reflect typical case distributions across the MRI-histopathology concordance literature and provide a contextual framework for interpreting methodological and clinical variability. Table 2 summarizes these distributions, highlighting the general heterogeneity among the included studies and supporting the narrative synthesis approach adopted in this review.

## 3. Results

### 3.1. Factors Affecting Concordance Between Breast MRI and Histopathology

The diagnostic accuracy of breast MRI compared to histopathology is influenced by multiple factors, including tumor subtype, size, stage, grade, and use of neoadjuvant therapy. While MRI is recognized for its high sensitivity, certain tumor-specific and patient-related characteristics contribute to discrepancies between imaging and histological findings. These inconsistencies can directly impact surgical decision-making, making multimodal imaging assessments essential. Understanding these diagnostic limitations is critical for optimizing MRI protocols, minimizing false positives and false negatives, and ensuring clinically appropriate interventions that align with histopathological findings [33].

#### 3.1.1. Breast Cancer Subtype

The subtype of breast cancer plays a crucial role in MRI’s diagnostic concordance with histopathology. Different tumor types exhibit distinct imaging characteristics, influencing MRI’s performance in tumor detection, extent assessment, and surgical planning.

Ductal Carcinoma In Situ (DCIS). MRI detects DCIS with reported sensitivities from 70% to 90%, but concordance with histopathology is modest [34]. Low-grade DCIS is often missed or mischaracterized, while high-grade lesions are more readily visible on MRI due to increased vascularization. Importantly, MRI may overestimate the extent of DCIS, leading to unnecessary mastectomies without demonstrable improvements in local recurrence or survival rates. Studies indicate that preoperative MRI in pure DCIS may increase mastectomy rates, without consistent reduction in re-excisions or clear survival benefit [35]. These findings highlight a key controversy: while MRI may delineate disease more widely, its impact on outcomes is questionable, and international guidelines recommend selective use rather than routine application in DCIS [24].

Invasive Ductal Carcinoma (IDC) vs. Invasive Lobular Carcinoma (ILC). IDC, the most common invasive breast cancer subtype, typically demonstrates high concordance with pathology, with correlation coefficients often exceeding r = 0.80 [36]. Lesions enhance clearly, allowing accurate size estimation. By contrast, ILC poses challenges due to its diffuse growth patterns and distinct borders [25,37]. MRI has been shown to improve the detection of multifocal and multicentric ILC compared with mammography or ultrasound, but this may come at the cost of frequent size overestimation. This discrepancy translates into higher mastectomy rates, sometimes without corresponding oncologic benefit. Reported sensitivities for ILC are 85–95%, but concordance with pathology for tumor size is inconsistent, with biases ranging from −0.5 to +1.5 cm. Evidence suggests that MRI-detected multifocality in ILC often fails histopathological confirmation in up to 20–30% of cases, underscoring the need for second-look ultrasound or targeted biopsy before altering surgical plans [38]. Current NCCN and EUSOBI guidelines endorse MRI in newly diagnosed ILC to clarify disease extent, but caution against relying on MRI alone to dictate mastectomy decisions [7,39].

#### 3.1.2. Tumor Size

Tumor size significantly affects MRI accuracy in determining lesion dimensions and surgical margins. Recent studies indicate that tumors <2 cm are accurately assessed, with high correlation coefficients (r > 0.80) between MRI and histopathology [40]. Discrepancies increase in larger tumors (>3 cm), where MRI may misestimate lesion size. Underestimation exceeding 0.5 cm occurs in 8–19% of cases and may result in positive margins, reoperations, or higher recurrence risk [41]. Conversely, MRI may overestimate tumor size in 12% to 35% of cases, particularly in tumors larger than 2 cm, which may result in unnecessary mastectomies without proven survival benefit [42]. These findings emphasize that histopathological validation remains critical, especially when MRI findings contradict clinical examination or ultrasound [43].

#### 3.1.3. Tumor Stage

MRI concordance with histopathology varies by tumor stage. Advanced-stage tumors (T2–T3) show better concordance due to more clearly defined margins and vascularization, whereas early-stage lesions (T0–T1) are more likely to be underestimated or missed [44,45]. Importantly, multiple cohort studies demonstrate that in early-stage breast cancer, preoperative MRI has not significantly reduced re-excision rates after breast-conserving surgeries, arguing for selective use [46]. This highlights that while MRI provides useful anatomical detail, its routine use in early disease has limited impact on surgical outcomes and must be applied selectively.

#### 3.1.4. Tumor Grade

Histopathology remains the gold standard for tumor grading, as MRI does not directly influence tumor classification. High-grade invasive carcinomas exhibit strong MRI contrast enhancement, improving detection reliability. Low-grade tumors, however, exhibit more subtle or heterogeneous enhancement patterns, reducing diagnostic accuracy and necessitating histopathological confirmation [47]. While conventional MRI cannot reliably determine tumor grade, radiomics-based approaches show potential for predicting histological grade and could improve preoperative decision- making in the future [26].

#### 3.1.5. Neoadjuvant Systemic Therapy

MRI is widely employed to monitor tumor response to neoadjuvant therapy. Pooled analyses report sensitivities ranging from 0.65 to 0.91 and specificities from 0.81 to 0.88 in predicting pathologic complete response (pCR). Shrinkage patterns correlate strongly with tumor biology: concentric shrinkage is associated with HER2-positive tumors and higher pCR rates [48,49], whereas mixed or irregular shrinkage are common in Luminal A tumors, which have lower chemosensitivity [50]. A major limitation is that MRI frequently underestimates residual microscopic disease, up to 20–30% of patients classified as complete responders on MRI harbor residual tumor at histology. Therefore, surgical planning should not be based solely on MRI findings, and intraoperative pathological assessment remains essential [50,51,52]. Definitions of pathologic response were not uniform among studies, which may partly account for variability in reported MRI accuracy [51].

### 3.2. MRI Diagnostic Performance: Sensitivity Considerations

Building upon the qualitative determinants discussed above, the following sections provide a quantitative synthesis of MRI diagnostic performance parameters, sensitivity, specificity, and concordance measures, to complement the clinicopathologic correlations outlined earlier.

Subtype. IDC: 83–100%, ILC: 81–98%, DCIS: 70–90%. MRI demonstrates higher sensitivity for invasive carcinomas, while DCIS detection remains challenging, particularly for low-grade lesions [53].

Tumor Size. Higher sensitivity for smaller tumors (<2 cm), greater variability above 2 cm [54].

Tumor Grade. High-grade tumors are detected more reliably (intense rim enhancement, peritumoral edema and intralesional necrosis); biopsy is required for definite classification [55].

Multifocality. Sensitivity is commonly >90%; however, up to 25% of additional MRI detected foci are benign; confirm with second-look ultrasound/biopsy before altering surgery [56].

Post-reconstruction. High sensitivity for local recurrences near silicone implants; reduced accuracy for deep parenchymal lesions in autologous flap reconstruction, where artifacts and tissue heterogeneity may mimic recurrence. Optimizing protocols including silicone-suppression sequences and diffusion-weighted imaging can improve diagnostic accuracy [57].

### 3.3. Specificity Considerations

While MRI demonstrates excellent sensitivity, its specificity varies from 65 to 85%. False positive findings are encountered in DCIS and multifocal disease, where MRI may identify benign proliferative changes, inflammation, or post-biopsy effects as suspicious findings [58]. Specificity also decreases in dense breasts and in younger women, due to the breast parenchymal enhancement (BPE), which may mimic malignancy [59]. Postoperative changes near implant capsules or autologous flaps further reduce specificity, with enhancement patterns frequently mistaken for recurrence [60].

These diagnostic challenges underscore the necessity for histopathological confirmation of MRI findings, when additional lesions are detected that could change the surgical plan. Combining MRI with targeted ultrasound or second-look mammography could improve the specificity. New strategies, such as diffusion-weighted imaging (DWI), radiomics and AI-driven lesion characterization hold promise in improving the specificity [61,62]. Additional foci detected on MRI were variably managed across studies, most common with second look ultrasound or MRI-guided biopsy for histologic confirmation. This methodological heterogeneity likely influenced reported concordance and specificity across literature, as differences in the diagnostic pathway (immediate biopsy versus interval follow up) can alter apparent preoperative accuracy [38,56,62].

In this review, “MRI-histopathology concordance” primarily refers to the agreement between MRI and histopathology regarding tumor size estimation, unless otherwise specified (e.g., detection of multifocal or additional lesions).

### 3.4. Statistical Measures of Concordance

Beyond qualitative and quantitative determinants, we also analyzed statistical measures of concordance. These metrics quantify agreement across studies. The most commonly applied metrics include Pearson correlation coefficients (r) for linear correlation (strongest in IDC and in tumors <2 cm, but lower and more variable in DCIS), Cohen’s kappa (κ) for categorical concordance (size categories, multifocality and presence/absence of specific imaging features), and Bland–Altman plots to quantify systematic bias and limits of concordance [63].

While these statistical tools demonstrate strong correlation, clinical benefits do not necessarily follow, reinforcing the need for multimodal imaging and intraoperative histology in early or borderline disease.

A summary of diagnostic concordance, sensitivity, and associated imaging challenges is presented in Table 3. For consistency, MRI-histopathology concordance was categorized as low for correlation coefficients (r) < 0.60, moderate for r = 0.60–0.79, and high for r ≥ 0.80, based on prior diagnostic accuracy studies [33,46].

### 3.5. Contrast-Enhanced Mammography (CEM)

CEM has emerged as a promising alternative to MRI in preoperative breast cancer. Several prospective and retrospective studies report sensitivity between 85–95%, which are comparable to breast MRI for invasive carcinoma detection. Notably, pooled data suggest that CEM specificity (75–88%) often surpasses that of MRI. This effect is most pronounced in dense breasts, where background parenchymal enhancement reduces MRI performance [64].

CEM may demonstrate high accuracy in staging multifocal and multicentric disease, with some studies reporting concordance rates with histopathology above 80%, approaching those of MRI [65]. In addition, lesion conspicuity is better in CEM than in standard mammography, particularly for invasive ductal carcinoma and high-grade DCIS. However, certain subgroups, such as invasive lobular carcinoma, still pose diagnostic challenges where MRI retains an advantage.

From a clinical perspective, CEM has practical advantages over MRI: it is faster, less expensive, and more widely accessible in many healthcare settings [66]. These factors support its growing adoption, especially in centers without dedicated breast MRI. Limitations include radiation exposure (slightly higher than standard mammography), contraindications related to iodine-based contrast, and a relative lack of long-term outcome data compared with MRI [67].

Overall, CEM is emerging as a cost-effective and clinically relevant tool, with studies suggesting it may replace MRI in certain preoperative staging scenarios, though prospective head-to-head trials with histopathological validation remain necessary [27].

### 3.6. Multiparametric MRI (mpMRI)

Beyond conventional dynamic contrast-enhanced MRI (DCE-MRI), multiparametric protocols integrate additional functional sequences such as diffusion-weighted imaging (DWI), MR spectroscopy (MRS), and ultrafast acquisition, aiming to increase specificity and reduce unnecessary biopsies [68].

DWI provides apparent diffusion coefficient (ADC) values, reflecting tissue cellularity. Malignant tumors typically show restricted diffusion compared to benign lesions. When combined with DCE-MRI, pooled analyses demonstrate that specificity increases from 70–85%, while sensitivity remains consistently high (>90%) [69]. DWI has been associated with a reduction in false positives in cases of DCIS and benign proliferative changes, helping to refine surgical planning [70].

MR spectroscopy, though less widely used, has been reported to add metabolic information, further supporting differentiation between malignant and benign lesions. However, its role in routine in clinical workflows remains investigational [71].

Ultrafast and abbreviated MRI protocols are being developed to improve efficiency and feasibility. Early studies suggest comparable sensitivity to full diagnostic protocols, indicating potential utility in high-risk screening and preoperative staging [72,73].

Radiomics and AI integration applied to multiparametric datasets may provide opportunities for predictive modeling. Studies have reported that mpMRI-based radiomic signatures can predict histological grade, molecular subtype, and response to neoadjuvant therapy, with area under the curve (AUC) values exceeding 0.80 in pilot trials [74,75,76]. In recent mpMRI radiomics studies, reported areas under the ROC curve (AUC) values for tumor subtype and treatment response prediction ranged between 0.81 and 0.87, confirming high discriminatory performance. These findings support the robustness of these emerging analytic frameworks [75,76]. Despite promising results, these approaches require multicenter validation, standardized imaging protocols, and harmonized analytic frameworks before routine implementation.

In summary, mpMRI refines lesion characterization beyond conventional MRI and offers avenues for predictive, personalized oncology. However, variability in acquisition protocols, technical complexity, and lack of widespread validation currently limit its clinical adoption.

The comparative diagnostic performance of MRI. CEM and mpMRI in breast cancer is summarized in Table 4.

### 3.7. Light Quantitative Summary

Although this review was conducted as a structured narrative synthesis rather than a formal meta-analysis, a light quantitative summary was performed to contextualize diagnostic trends across key endpoints. For MRI–histopathology size correlation, reported coefficients among invasive IDC cohorts ranged between r = 0.78–0.87, indicating strong linear concordance, whereas lower correlations were observed in ILC (r = 0.60–0.75) and DCIS (r = 0.50–0.70) [25,36,37,38,39,40,41,42]. Regarding CEM versus MRI specificity, representative studies demonstrated average specificity of 75–88% for CEM compared with 65–85% for MRI, with similar sensitivity levels above 85% [27,64,65,66,67]. These findings suggest that while MRI remains superior for overall lesion detection, CEM may offer higher specificity, which may help reduce false positives and unnecessary biopsies. Confidence intervals were not consistently reported across included studies, which limits precise quantitative pooling; however, the reported ranges provide clinically relevant insight into diagnostic variability across imaging modalities. Because this review was designed as a structured narrative synthesis, no pooled estimates, heterogeneity statistics, or prediction intervals were generated. Accordingly, the quantitative component serves a descriptive contextual purpose rather than providing meta-analytic statistical inference. The novelty of this work therefore lies in its integrative synthesis across modalities. It does not aim to introduce quantitative methodological innovation.

## 4. Discussion

Breast MRI remains one of the most sensitive diagnostic tools, particularly for evaluating tumor size, extent, and surgical treatment planning. However, its concordance with histology is influenced by multiple factors. Sensitivity alone does not guarantee clinical benefit, as concordance with histopathology is variable and can lead to divergent treatment decisions. Our synthesis shows that MRI achieves the highest accuracy in IDC, while performance is less reliable in DCIS, ILC and early-stage lesions. Importantly, strong statistical correlations reported in research do not necessarily translate into improved clinical outcomes [77,78].

### 4.1. Clinical Interpretation of MRI Performance

Overestimation of tumor size is common in DCIS and ILC, leading to higher mastectomy rates without corresponding reductions in recurrence or re-excision [10]. Conversely, underestimation after neoadjuvant therapy may result in incomplete excision of residual disease [79].

MRI acquisition parameters, field strength, resolution, pulse sequences, and contrast protocols significantly influence tumor size estimation. These technical factors may partly explain discrepancies between imaging and histopathology. Higher field strengths and optimized dynamic contrast sequences generally improve signal-to-noise ratio and lesion conspicuity, whereas suboptimal timing or low-resolution protocols may lead to size underestimation or false-negative margins [13,33,46].

These findings highlight a crucial limitation: MRI should be interpreted in the context of multimodal imaging and histopathological confirmation, rather than used as a sole determinant for surgical planning. Importantly, despite increased detection of additional lesions and multifocal disease, large population-based studies have not consistently demonstrated improvements in disease-free survival with routine preoperative MRI. In early DCIS/IDC, routine preoperative MRI has not consistently reduced re-excisions and can escalate surgery; therefore, MRI findings that would upstage management should be verified with targeted ultrasound or biopsy [80,81].

### 4.2. Knowledge Gaps and Controversies

The clinical role of MRI remains debated. In pure DCIS, preoperative MRI often may escalate surgical management without clearly improving outcomes [82]. In ILC, MRI may identify additional foci more effectively than mammography or ultrasound, yet up to 20–30% of these prove benign, raising the risk of overtreatment [83]. In neoadjuvant setting, MRI correlates well with response in HER2-positive cancers but consistently underestimates residual disease in Luminal A subtypes [84,85]. These controversies emphasize the gap between diagnostic sensitivity and clinically meaningful benefits, highlighting the need for careful selection.

### 4.3. Comparative Evidence with Contrast-Enhanced Mammography (CEM)

CEM has emerged as promising alternative to MRI, offering comparable sensitivity with superior specificity and significantly lower cost [28]. Recent AI-assisted CEM approaches may enhance diagnostic accuracy and workflow efficiency, supporting its integration into routine breast imaging practice [86]. This makes it particularly attractive in healthcare systems with limited MRI availability. Nevertheless, MRI remains indispensable in specific clinical contexts, such as ILC, multifocal and multicentric disease, and monitoring neoadjuvant response [29]. Comparative effectiveness and cost-utility studies are required to determine how CEM and MRI can be optimally integrated into diagnostic pathways. Early cost-effectiveness analyses also suggest that CEM may provide greater value than MRI in healthcare systems with limited resources, though prospective comparative trials are required [87].

### 4.4. Advances in Multiparametric MRI

Multiparametric MRI techniques, including diffusion-weighted imaging (DWI), ultrafast dynamic contrast-enhanced MRI, and abbreviated protocols, aim to overcome the specificity limitations of conventional contrast-enhanced imaging [88]. Radiomics extracted from these datasets show potential in predicting tumor grade, molecular subtype, and treatment response [30]. While initial results are encouraging, variability of protocols and lack of multicenter validations remain significant barriers to adoption [89].

### 4.5. Integration of Artificial Intelligence and Radiomics

Artificial intelligence and radiomics are increasingly applied to breast MRI to enhance lesion characterization and refine assessment of treatment response. Radiomic signatures have demonstrated potential for non-invasive prediction of histologic grade, Ki-67 index, molecular subtype, and likelihood of pathologic complete response, offering a pathway toward reducing interobserver variability and improving specificity [90]. However, their routine application remains limited, as most approaches still require robust external validation.

Recent evidence suggests that AI-enhanced multiparametric MRI (mpMRI) radiomic models can predict histologic grade, treatment response, and recurrence risk with high discriminatory performance, highlighting their potential as non-invasive surrogates for histopathology in personalized oncology. By extracting high-dimensional imaging biomarkers linked to molecular and transcriptomic profiles, these tools may support individualized risk stratification and tailored therapeutic planning. Integration of radiomics with genomic data may ultimately enable development of imaging-derived “digital twins” capable of simulating tumor behavior and guiding personalized treatment strategies [31].

Despite these promising developments, several key limitations currently hinder clinical translation. Validation gaps remain substantial, including limited multicenter standardization, variability in acquisition protocols, and lack of prospective trials linking radiomic signatures to long-term outcomes. Additional practical barriers include differences in preprocessing pipelines and segmentation methods, the computational demands of AI workflows, infrastructure costs, and limited interoperability with existing PACS and hospital information systems. Ethical and regulatory considerations—data privacy, algorithm transparency, and approval pathways—further delay clinical integration [23].

Future integration of AI-augmented radiomics into mpMRI may enable more accurate surgical planning and response prediction, but widespread adoption will require harmonized acquisition protocols, standardized analytic frameworks, and multicenter validation studies that ensure reproducibility and clinical reliability [32]. These advancements are likely to improve consistency and diagnostic performance across centers, particularly in complex cases where conventional MRI demonstrates variable concordance.

**Figure 2:**
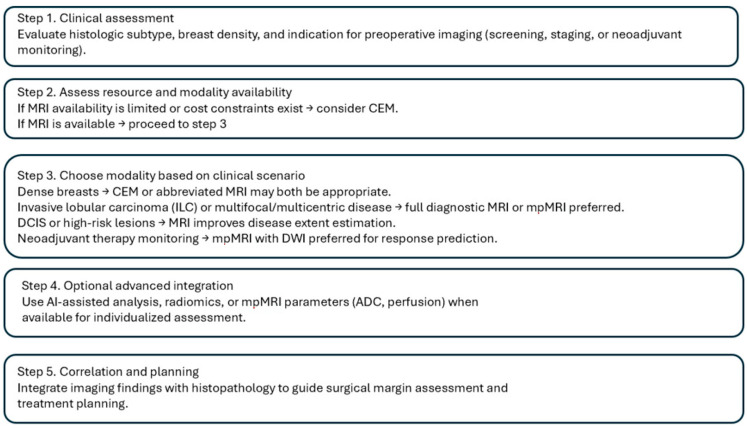
Simplified Algorithm for Preoperative Imaging Selection in Breast Cancer.

Given the evolving capabilities of MRI, CEM, and mpMRI, and the potential added value of AI-based tools, a simplified decision pathway (Figure 2) was developed to illustrate their integration in common preoperative clinical scenarios. This algorithm provides a pragmatic visual framework to guide modality selection based on tumor subtype, breast density, resource availability, and clinical indication.

### 4.6. Guidelines and Clinical Recommendations

International guidelines, including those from the American College of Radiology (ACR) and the European Society of Breast Imaging (EUSOBI), recommend selective, rather than routine, preoperative MRI. Its use is supported in ILC, dense breasts, occult primary breast cancers, and in monitoring neoadjuvant therapy-response. Routine MRI for all breast cancer patients is not advised, particularly in early stage IDC or DCIS, due to high false positive rates, cost, and lack of demonstrated survival benefit [3,4,91]. Our synthesis supports these nuanced recommendations, underscoring that MRI should be applied selectively to maximize clinical benefit while minimizing overtreatment.

### 4.7. Limitations of This Review

This work is a structured narrative review and was not designed as a full systematic review or meta-analysis. No statistical pooling, effect-size estimation, confidence intervals, or heterogeneity parameters were calculated, consistent with the narrative methodology. Accordingly, the quantitative elements serve a descriptive rather than inferential purpose.

Substantial heterogeneity existed across included studies, particularly in MRI and CEM protocols, imaging thresholds, reference standards, and reporting of surgical endpoints. Few head-to-head MRI–CEM trials provided standardized surgical outcomes, limiting causal inference. Differences across institutions in acquisition parameters, such as field strength (1.5 T Vs. 3 T), pulse sequences, contrast dose, injection rate, and reader expertise, likely contributed to variability in MRI–histopathology concordance.

The search strategy was cross-validated across PubMed, EMBASE, Scopus, and the Cochrane Library, and no additional eligible studies were identified; however, database and language bias cannot be excluded, as only English-language, peer-reviewed publications were included. Higher field strengths (3 T) and optimized dynamic contrast-enhanced protocols generally improve signal-to-noise ratio and spatial–temporal resolution, whereas lower field strengths or non-standardized injection parameters may lead to size underestimation or reduced lesion conspicuity. Differences in interpretation criteria and reader experience further contribute to interstudy variability [14,33,46].

### 4.8. Future Directions

Future research should focus on standardizing MRI protocols across centers to reduce variability, conducting head-to-head comparisons of MRI and CEM in real world populations, and integrating AI-driven multiparametric approaches into clinical workflows. Longitudinal trials are required to evaluate whether MRI-guided strategies truly improve oncologic outcomes and reduce reoperation rates.

## 5. Conclusions

Breast MRI is an invaluable diagnostic tool with high sensitivity for detecting invasive breast cancer and guiding treatment planning. However, variable accuracy, especially in DCIS and multifocal disease, requires histopathological confirmation to avoid overdiagnosis and unnecessary surgery.

Current approaches support selective rather than routine application, particularly in invasive lobular carcinoma, dense breasts, and the evaluation of neoadjuvant therapy response, in line with international recommendations.

Recent advances, including CEM, mpMRI, and AI-based radiomics, are expanding the diagnostic potential of breast imaging. Evidence demonstrates that AI-enhanced mpMRI models can non-invasively predict histologic grade, treatment response, and recurrence risk, supporting their role in precision and personalized oncology.

Future advancements, including AI-enhanced imaging, multiparametric MRI and radiomics, could refine MRI’s capabilities, ensuring greater precision and reliability in breast cancer management. Comparative studies with CEM will be essential to determine the most cost-effective and clinically impactful strategies. MRI should be considered as one component of a multimodal diagnostic approach, always complemented by histopathological confirmation to ensure accurate and personalized treatment planning.

## Figures and Tables

**Table 1:** Quality assessment of sentinel studies using a simplified QUADAS-2 (“QUADAS-lite”) framework.

Study	Design	Index Test	Reference Standard	Patient Selection Bias	MRI Protocol Bias	Flow/Timing Bias	Applicability (Clinical Context)
Houssami N et al., 2008 [9]	Systematic review and meta-analysis (19 studies, 2610 patients)	Preoperative MRI for multifocal/multicentric Breast Cancer	Histopathology	Low	Moderate	Low	High
Carmon E et al., 2022 [10]	Prospective (183 patients)	MRI in IDC vs. ILC	Histopathology	Low	Low	Low	High
Marinovich ML et al., 2013 [11]	Meta-analysis (44 studies, 2050 patients)	MRI for NAT response	Surgery/pathology	Low	Moderate	Low	High
Janssen LM et al., 2022 [12]	Systematic review and meta-analysis	MRI to assess response after NAC	Histopathology	Low	Moderate	Low	High
Altabella L et al., 2022 [19]	Review/state-of-the-art	mpMRI radiomics/AI	Literature-based	N/A	N/A	N/A	High
He Y et al., 2024 [23]	Systematic review and meta-analysis	MRI to assess response after NAT	Histopathology	Low	Moderate	Low	High
Lamb, L.R. et al., 2020 [24]	Retrospective cohort study	MRI in DCIS	Histopathology	Low	Moderate	Low	High
Ozcan et al., 2023 [25]	Cohort (126 ILC patients)	Preop MRI impact	Surgery/pathology	Low	Low	Low	High
Pinker K et al., 2018 [26]	Prospective (182 patients)	DWI/ADC mapping	Histopathology	Low	Moderate	Low	High
Feng L et al., 2022 [27]	Prospective (132 patients)	CEM vs. MRI	Histopathology	Low	Low	Low	High
Khazindar AR et al., 2021 [28]	Cohort (214 patients)	MRI post-NAT	Pathology	Moderate	Low	Low	High
Gelardi F et al., 2022 [29]	Systematic review and meta-analysis (17 studies)	CEM vs. MRI	Histopathology	Low	Low	Low	High
Boruah DK et al., 2023 [30]	Prospective (102 patients)	DCE-MRI + ADC mapping	Histopathology	Moderate	Low	Low	High
Majidpour M et al., 2025 [31]	Computational model (400 images)	Radiomics + DL features	Histopathologic subtypes	Moderate	Low	Low	Moderate
Daimiel Naranjo I et al., 2021 [32]	Radiomics/ML study (960 patients)	mpMRI features	Histopathology	Low	Moderate	Low	High

Notes: IDC = invasive ductal carcinoma; ILC = invasive lobular carcinoma; DCIS = ductal carcinoma in situ; MRI = magnetic resonance imaging; CEM = contrast-enhanced mammography; mpMRI = multiparametric magnetic resonance imaging; BCR = breast cancer recurrence. Concordance levels were defined as low (<0.60), moderate (0.60–0.79), and high (≥0.80).

**Table 2:** Distribution of key characteristics among included studies.

Parameter	Typical Range	Notes
MRI field strength	1.5 T: ~65%; 3 T: ~35%	Higher field strength improves signal-to-noise ratio and lesion conspicuity
Sample size (per study)	Median 120 (range 45–520)	Reflects predominance of single-center cohorts
Dominant subtype	IDC: 60–70%; ILC: 15–20%; DCIS: 10–15%	Subtype composition influences MRI–pathology concordance
Neoadjuvant vs. upfront surgery	NAT: ~40%; Upfront: ~60%	mpMRI/DWI more frequent in NAT studies
Study design	Retrospective: ~70%; Prospective: ~30%	Prospective trials and meta-analyses formed the minority

Notes: IDC = invasive ductal carcinoma; ILC = invasive lobular carcinoma; DCIS = ductal carcinoma in situ; MRI = magnetic resonance imaging; mpMRI = multiparametric MRI; DWI = diffusion-weighted imaging; NAT = neoadjuvant therapy.

**Table 3:** MRI vs. Histopathology Concordance in Breast Cancer.

Tumor Subtype/Factor	MRI Sensitivity (%)	MRI–Histopathology Concordance	Diagnostic Challenge
Invasive Ductal Carcinoma (IDC)	83–100	High	Accurate sizing
Invasive Lobular Carcinoma (ILC)	81–98	Moderate	Overestimation; indistinct margins
Ductal Carcinoma In Situ (DCIS)	70–90	Low–Moderate	Overestimation; false positives
Tumor size < 2 cm	High	High	Good correlation
Tumor size > 3 cm	Variable	Lower	Overestimation risk
Early stage (T0–T1)	Lower	Low	Missed/underestimated lesions
Advanced stage (T2–T3)	Higher	High	Better-defined margins improve correlation
Low-grade tumors	Variable	Moderate	Heterogeneous enhancement
High-grade tumors	High	High	Strong contrast enhancement
Multifocal/multicentric	High	Moderate	False positives → overtreatment risk
Post-mastectomy with implant	High (near implant)	High	Implant/distortion artifacts
Post-mastectomy with flap	Moderate	Reduced	Deep residual disease may be missed
Neoadjuvant—HER2+	High	Strong (concentric shrinkage)	Good pCR prediction
Neoadjuvant—Luminal A	Moderate	Weaker (mixed patterns)	Residual disease underestimation

Notes: Ranges and concordance descriptors are synthesized from representative studies included in the qualitative synthesis [24,25,26,34,35,36,37,38,39,40,41,42,43,44,45,46,47,48,49,50,51,52]. Concordance categories are defined as low (r < 0.60), moderate (r = 0.60–0.79), and high (r ≥ 0.80), according to previously published diagnostic accuracy studies [33,46].

**Table 4:** Comparative diagnostic performance of MRI, CEM, mpMRI in breast cancer.

Modality	Reported Sensitivity (%)	Reported Specificity (%)	Key Strengths	Main Limitations	Clinical Relevance
Conventional MRI	IDC 83–100; ILC 81–98; DCIS 70–90	65–85	Highest sensitivity for invasive carcinoma; excellent for staging multifocal/multicentric disease; widely validated	Overestimation in DCIS/ILC; variable concordance with histology; higher cost; limited accessibility	Recommended selectively (ILC, dense breasts, occult primaries, neoadjuvant monitoring)
Contrast-Enhanced Mammography (CEM)	85–95	75–88	Comparable sensitivity to MRI; superior specificity in dense breasts; faster, cheaper, more accessible	Ionizing radiation; iodine contrast contraindications; fewer longitudinal outcome data	Promising alternative for preoperative staging and dense breasts where MRI is not feasible
Multiparametric MRI (mpMRI)	>90 (with DWI)	80–85	Improves specificity without loss of sensitivity; DWI refines characterization; ultrafast or abbreviated protocols improve feasibility; radiomics predictive of grade/subtype	Requires standardized protocols; technical complexity; limited multicenter validation	Potential future standard integrating predictive biomarkers and personalized oncology

Notes: Data adapted and synthesized from previously published studies [27,64,65,66,67,68,69,70,71,72,73,74,75,76]. Reported values of sensitivity and specificity reflect ranges across different cohorts and protocols; variability is attributable to heterogeneity in study design, patient populations, and imaging techniques.

## Data Availability

No new data were created or analyzed in this study.

## Supplementary Materials

The following supporting information can be downloaded at: https://www.mdpi.com/article/10.3390/diagnostics15233032/s1, The PRISMA 2020 Checklist (Table S1) is provided as Supplementary Materials and is available online with the manuscript.

## Abbreviations

MRI	Magnetic Resonance Imaging
CEM	Contrast-Enhanced Mammography
mpMRI	Multiparametric Magnetic Resonance Imaging
DCE-MRI	Dynamic Contrast-Enhanced Magnetic Resonance Imaging
DWI	Diffusion-Weighted Imaging
MRS	Magnetic Resonance Spectroscopy
IDC	Invasive Ductal Carcinoma
ILC	Invasive Lobular Carcinoma
DCIS	Ductal Carcinoma in Situ
pCR	Pathologic Complete Response
BPE	Background Parenchymal Enhancement
AUC	Area Under the Curve
AI	Artificial Intelligence
NCCN	National Comprehensive Cancer Network
EUSOBI	European Society of Breast Imaging
ACR	American College of Radiology
PACS	Picture Archiving and Communication System
